# Diagnostic formulation

**DOI:** 10.4103/0019-5545.58905

**Published:** 2010

**Authors:** K. Kuruvilla, Anju Kuruvilla

**Affiliations:** Department of Psychiatry, PSG Institute of Medical Sciences and Research, Coimbatore, India; 1 Department of Psychiatry, Christian Medical College, Vellore, India

**Keywords:** Diagnostic formulation, post-graduate examination, guidelines

## Abstract

Writing a ‘Diagnostic Formulation’ is a skill expected of candidates in the post-graduate examinations in psychiatry in most universities in India. However there is ambiguity regarding what the term means and how it should be written. This article is an attempt to provide some guidelines on this topic.

## INTRODUCTION

One of the great attractions of psychiatry is the considerable scope for disagreement-on every aspect including diagnosis, aetiology, treatment and the very nature of psychiatric disorders. It is presumably because of this that the process of formulation has evolved-a process by which the features of an individual case can be discussed and evaluated to consider a series of possibilities, which will guide the management. This seems to be an admirably flexible way to deal with the complexities and uncertainties of psychiatric diagnosis[1] and so has become a skill, which is given great importance in the qualifying examinations in psychiatry in many countries.

## WHAT IS A DIAGNOSTIC FORMULATION?

In a survey, examiners of the Royal College of Psychiatrists were asked to give their opinion on why candidates fail the membership clinical examinations. About 87% opined that the major cause of failure was ‘poor diagnostic formulation’. Issues like ‘lack of factual knowledge’ (40%) and ‘lack of clinical experience’ (26%) were rated a distant 2^nd^ and 3^rd^.[2]

There is however disagreement about what the term ‘formulation’ implies and what it includes. Psychiatrists at various levels of seniority working in a teaching hospital were asked to list what should be included in a diagnostic formulation.[3] The results are shown in Table 1.

**Table 1:** Components of diagnostic formulation - Opinion among psychiatrists

Item	Consultants	Sr. Reg	Reg./SHO	Total sample
History (%)	81	82	81	81
Mental status	57	64	38	52
Physical exam	10	18	13	13
Diff. diagnoses	76	64	50	65
Single diagnosis	24	36	37	31
Psychodynamics	33	45	19	31
Aetiology	43	45	56	48
Prognosis	67	55	69	65
Length of formulation	29	0	44	27

(Sr. Reg - Senior registrar; Reg. - Registrar; SHO - Senior House Officer)

The following three points are striking-firstly, differential diagnosis is preferred to single diagnosis as seniority increases. Secondly, psychodynamics was considered important by only one-third of respondents-least of all by juniors-perhaps because they understood the term as related to psychoanalytic concepts. The authors however had used the term to refer to ‘psychological and emotional factors surrounding the patient’. Thirdly, physical examination was not given importance by any group.

A similar survey was conducted with examiners of MRCPsych Part II clinical exam.[4] The results showed that even among examiners, there is no consensus on what a formulation should contain [Table 2].

**Table 2:** Components of diagnostic formulation - Opinion among examiners

Item	Examiners%
History	78
Mental status	62
Physical exam	07
Investigations	49
Diff. diagnoses	80
Single diagnosis	11
Psychodynamics	29
Aetiology	60
Management	82
Prognosis	69
Length of formulation	51

There are conspicuous differences in the opinion of the examiners as compared to that of the prospective candidates with respect to the important components of a diagnostic formulation [Table 3].

**Table 3:** Components of diagnostic formulation - Opinion among examiners and trainees

Item	Examiners%	Trainees%
Mental status	62	38
Investigations	49	13
Diff. diagnoses	80	50
Single diagnosis	11	37

When there are significant discrepancies between the examiner's expectations and the candidate's assumptions regarding the importance of items like mental status, differential diagnoses, investigations etc., in a diagnostic formulation, the candidate's performance will very likely be viewed as ‘poor’ by the examiner. Lack of consensus on many aspects of the diagnostic formulation also leads to comments like -'this is a summary, not a formulation', ‘the formulation does not include management’, ‘give us your formulation in two minutes, please’. Such comments are not only anxiety provoking for the candidate but also reflect an unnecessarily rigid point of view. Instead, it is more appropriate to provide flexible guidelines on formulation for examiners and candidates, in order to facilitate a discussion about the patient and his problems, which after all, is the purpose of a clinical examination.

## WHAT ARE THE GUIDELINES ON DIAGNOSTIC FORMULATION?

The Institute of Psychiatry guidelines[5] to candidates on eliciting and recording clinical information, refers to an ‘Initial Formulation’ and defines it as ‘a doctor's assessment of the case rather than a re-statement of facts. Its length, layout and emphasis will vary considerably from one patient to another. It should always include the following: (1) a discussion on the diagnosis (2) aetiological factors, which seem important, as well as taking into account (3) the patient's life situation and background, with (4) a plan for treatment and (5) an estimate of the prognosis. Regardless of the uncertainty or complexity of the case a Provisional Diagnosis should always be specified’.

The Association of Psychiatrists in Training (APIT)[5] defined diagnostic formulation as ‘An account of deductions based on data obtained from the history and examination, followed by management plans’. It recommended that the diagnostic formulation include (i) A brief two or three line‘Introduction’ stating the problem, for example, ‘This is a 35-year-old housewife whose main complaint is that she has been unable to leave her home for the past two years’; (ii) Differential diagnoses, e.g. agoraphobia/depressive illness; (iii) Justification-reasons for the differential diagnoses including positive points from the history and mental state examination, which make one diagnosis more likely than the other; (iv) Further investigations-history from informants, old case notes, relevant physical and psychological investigations; (v) Management plans-admission to hospital, medication, psychological treatment, family involvement, rehabilitation, long-term treatment and (vi) Prognosis.

Varghese and Mellsop[6] reported that ‘formulation’ was considered an important part of case writing in the FRANZCP examinations. Twenty four examiners of the Royal Australian and New Zealand College of Psychiatrists were asked to rate twelve diagnostic formulations. Correlation between the ratings of the examiners was high. High ratings were given to formulations, which were considered to be ‘concise, comprehensive and well set-out without jargon’. Formulations were rated to be poor on the basis of ‘inadequate data (history and MSE), unjustified diagnosis and differential diagnoses and unjustified treatment’. According to these authors, a good formulation should include ‘a meaningful summary and sufficiently defensible diagnostic statements that should lead to the development of a coherent and planned approach to management. If a statement on aetiology is made, especially if a psycho dynamic understanding is attempted, this should be stated in ordinary language, avoiding jargon. It should rely on information presented in the case and not on some presumed theory of aetiology’.

The Oxford Textbook of Psychiatry[7] states that ‘formulation is a concise statement of the case. Unlike summary, it is a discussion about alternative ideas about diagnosis, aetiology, treatment and prognosis and of the arguments for and against each alternative. A good formulation is based on the facts of the case and not on speculation. It may contain verifiable hypotheses about matters that are uncertain at the time of writing. A formulation is concerned with not only the disease concepts, but also with the understanding how the patient's lifelong experiences have influenced his personality and his ways of reacting to adversity’. The New Oxford Textbook of Psychiatry[8] considers diagnostic formulation essentially as ‘an analysis and integration of information’.

Sims and Curran[9] opine that ‘formulation’ is a concise review of the case and state that ‘A good formulation is a balanced appraisal of the psychiatric assessment, but should be very firmly based on the facts of the case rather than on speculation. It also encapsulates the main issues of the case, which facilitate communication with other professionals’.

## HOW IS A DIAGNOSTIC FORMULATION CONSTRUCTED?

### General principles

1. Formulation is about an individual patient. General psychiatric knowledge should be introduced only when relevant to the particular case.
2. It should not sound like a textbook description of a psychiatric disorder.
3. There is a fairly well-accepted structure for the formulation - but one should be flexible in its use and adapt it to suit the individual patient. For example, if the patient is unable to give a good account of the presenting problem, more attention should be paid to the MSE, and during the discussion on management, emphasis should be on how to obtain further information.
4. The duration of the presentation of the diagnostic formulation should last about 5 minutes, but one should be prepared to increase or reduce it, depending on demand from examiners. Generally, the recommended length for a written version is one side of an A4-sized paper.

### Structure

Introductory comments: Present the salient socio-demographic features of the patient (e.g. ‘Mrs. R is a 30-year-old married school teacher living with her husband and a 4-year-old son’).

Presenting problems: This section should be brief; state the main problems excluding irrelevant details (e.g. ‘Over the past two months she has become increasingly depressed, with loss of energy, self-reproaches and self-depreciating ideas’).Briefly mention how the patient's life has been affected by the problems (e.g. ‘She has not been going for work and has also been unable to do the housework or take care of her child’).Mention events closely related to the onset or exacerbation (e.g. ‘The onset of symptoms was preceded by a medical termination of pregnancy about which patient was very ambivalent’). Avoid long lists of minor or transient symptoms and negative findings except those that will help in the differential diagnosis.

Past history of psychiatric disorder, its treatment and outcome: (e.g. ‘Mrs. R had had similar symptoms soon after her son's birth; she was treated with antidepressant medication and became completely well in about two months’).

Positive medical history of significance: (e.g. ‘The patient was detected to be hypothyroid a year ago and is on treatment’).

Mental status examination: Mention important findings only. Use labels for psychopathological findings at this stage, for example, use terms such as ‘delusions of guilt’, ‘third person auditory hallucinations’ etc. Details of these findings should have already been described during the detailed presentation prior to the formulation and if helpful, could be mentioned again during the discussion of the differential diagnoses.

Differential diagnosis: If there is little doubt about the diagnosis, say so and say why. Do not present an irrelevant differential diagnosis for the sake of giving one.

If diagnosis is not clear, embark on a careful discussion of the possibilities in the order of likelihood, and discuss points in favor of and against each of them. This is done using descriptive psychopathology (e.g. first-rank symptoms) elicited during history taking and mental status examination. Details of symptoms collected earlier could be used to support a diagnosis (e.g. content of auditory hallucinations to differentiate between schizophrenia and depressive illness). Information on the course of illness is also useful (e.g. ‘Though the acute psychotic symptoms are remitted with medication, the patient never reached his premorbid level of functioning at work or in social interactions’).

Differential diagnosis tests one's ability to make a discriminating clinical judgment. Do not give a long list of differential diagnoses that cover the whole of ICD-10; think twice before giving more than three or four.

If a patient's history and findings justify diagnosing two or more conditions that co-occur, mention those with supporting evidence (e.g. Depressive disorder in a person with alcohol dependence syndrome). End the discussion with a conclusion on the most likely diagnosis. If that is not possible at all, mention the major possibilities.

Aetiological factors: These could be considered from different perspectives for example based on nature or based on chronology.

#### Nature

Biological Factors - e.g. Genetic, physical illness, drugs

Psychological Factors - e.g. Obsessive personality traits

Sociocultural Factors - e.g. Poor social support, unemployment

#### Chronological

Predisposing Factors - e.g. Family history of mood disorders

Precipitating Factors - e.g. Child birth

Perpetuating Factors - e.g. Husband‘s alcohol abuse

#### Management:

- Further investigations
  Includes information from key relatives/employer/teachers
  Review of past case records
  Laboratory investigations
  Psychometry
  In each case specify which procedure/tests you would organize and its justification
- Immediate management plans
  Is the patient to be treated as an inpatient or outpatient?
  If as an inpatient, why?
  Management of suicide risk/violence - where indicated Medication - specify type/justification/dosage/route/expected response/side effects and their managements.
- Long-term management plans
  - Somatic: Medication - type/dosage/duration
  - Psychological: Psychotherapy - indications/type/focus
  - Social: Involvement of the family/rehabilitation measure

Prognosis: This should not be a general pronouncement, based merely on the type of disorder (such as schizophrenia). Discuss instead the good (e.g. acute onset; affective symptoms) and poor (e.g. poor drug compliance in the past; poor social support) prognostic factors. Prognosis can also be described under the headings of short term (e.g. ‘Chances of recovery from the present episode is good with antidepressant treatment’) and long term (‘risk of relapse and recurrence is high because of the significant marital discord and patient's reluctance to take medicines on a long-term basis’). Come to a reasonably firm final conclusion rather than using vague terms like ‘guarded’.

## HOW IS A SUMMARY DIFFERENT FROM A DIAGNOSTIC FORMULATION?

The terms ‘summary’ and ‘diagnostic formulation’are often used together and cause confusion to many candidates who take them to be synonymous. However, there are subtle but important differences and being aware of them is helpful in making a good diagnostic formulation.

Summary is a concise description of all the important aspects of the case, whereas formulation is an assessment of the case rather than a restatement of facts. The best example of a summary is the Discharge Summary, given on discharge after an inpatient treatment. This should be written in such a way that it provides all the necessary information that will assist in the follow-up care of the patient by the same, or other medical team. The summary should include:

- Demographic data like name, age, gender
- Reasons for referral to psychiatry
- History of present illness
- History of previous illnesses
- Family history
- Personal history - birth and development, childhood, education, occupation, sexual and marital history
- Premorbid personality
- Physical examination
- Investigations - physical and psychological
- Diagnosis
- Treatment and progress
- Prognosis'
- Plans for further management

## WHAT ARE THE DIFFERENT TYPES OF FORMULATIONS?

Form and content of a formulation will vary depending on the following:

Context: After a detailed presentation of the case, as it is usually done for a ‘long case’, it is generally not expected or necessary to give a summary before presenting the diagnostic formulation, unless circumstances warrant it.

Time for assessment: Initial assessment formulation (e.g. diagnostic formulation done at a clinical examination) differs from formulation of a well-investigated case (e.g. presentation in a case conference with fellow professionals), where more details about psychodynamics and management issues are given.

Type: Diagnostic formulation differs from psychodynamic formulation used in psychotherapeutic settings.[10]

Purpose: There should be difference between a formulation given at the end of a case presentation and a formulation expected to stand on its own. A good example of the latter is the Comprehensive Diagnostic Formulation[11–12]. It has two components, the Standardized multi-axial diagnostic formulation and the Personalized idiographic formulation.

### Standardized multi-axial diagnostic formulation

**Table:** 

a. Axis I:	This comprises mental disorders including personality disorders and developmental disorders as well as general medical conditions.
b. Axis II:	This addresses disabilities in (i) personal care (ii) occupational functioning (iii) functioning with family and (iv) social functioning. Each is rated on a scale of 0 (none) to 5 (massive).
c. Axis III:	Contextual factors. Problem areas such as housing, education, work, financial, legal and interpersonal are included here.
d. Axis IV:	Quality of life. This is scored from 1 (poor) to 10 (excellent) and reflects the patient's own perceptions.

### Personalized idiographic formulation

- Clinical problems and their contextualization. These include problems based on the standardized multi-axial formulation with complementary information from the biological, psychological, social and cultural perspectives.
- Positive factors of the patient. Factors relevant to the treatment and to health promotion, such as maturity of personality, skills, talents, social resources and supports.
- Expectation on restoration and promotion of health, about the treatment as well as aspiration about health and quality of life.

## DECLINE AND DEMISE OF DIAGNOSTIC FORMULATION IN EXAMINATIONS OF THE ROYAL COLLEGE OF PSYCHIATRISTS, U.K.

Because of the lack of consensus on what the term ‘formulation’ means, what it should include and how useful a concept it is, the term was dropped in the mid-1980s by the Royal College, and candidates were asked to give an ‘assessment’ of the long case consisting of relevant factors from the history, mental status examination and physical examination. Candidates were expected to make appropriate deductions from the information given, present differential diagnoses, management plans and prognosis.[13]

The ‘long’ case, which had been the cornerstone of the clinical examination in Part II MRCPsych examinations, has been criticized as an imperfect method of assessment of a candidate's clinical abilities because cases given to different candidates cannot be standardized for diagnostic complexity, cooperation of the patient, examiner's style of questioning etc. The assessment becomes extremely unreliable and some studies have shown that the reproducibility co-efficient of the marks achieved in the long case is as low as 0.24. Therefore in the year 2008, the Observed Structured Clinical Examination (OSCE) replaced the long case. Along with the long case, the ‘formulation’ - long venerated, disputed and debated, has also been dispensed with.[14]

There are many who question the wisdom of this reform. It is argued that the long case represents psychiatry's commitment to a holistic approach and so discarding it from the examination system will lead to a failure to test the ability of the candidate to integrate and synthesize all the information obtained from an interview with the patient - and ultimately this will lead to the discarding of the bio-psycho-social model.[15]

## A MEMORY AID FOR DIAGNOSTIC FORMULATION

Whitley[16] has suggested a mnemonic for the components of a diagnostic formulation:

Facts of life – Summary of patient's socio-demographic details

Onset of illness – Nature of onset and past history of illness

Recent mental illness – Current episode and important symptoms

### Mental state

Umpteen diagnoses – Differential diagnoses

Lack of information – How to compensate for it through interview of relatives, investigations etc.

Aetiology – Predisposing, precipitating and perpetuating factors

### Treatment

Inpatient management – Justification of the need, expected contributions from other professionals like nurses, occupational therapists etc.

Outcome – Short-term and long-term prognosis

New attacks of illness – How to prevent them (e.g. mood stabilizers, family education)?

### Some dos and don'ts

1. Learn to make a logical system of formulation; practice it by doing it for every case, at least in the last year of your post graduation course.
2. Plan your time to have enough to think and write out the formulation.
3. Remember that no reasonable examiner will insist on a single firm diagnosis (unless of course, you get an absolutely typical, ‘text book’ case).
4. Do not make the examiners work hard to ‘extract’ information from you.
